# DEPIGMENTATION ALONG LYMPHATIC CHANNELS FOLLOWING INTRALESIONAL CORTICOSTEROID INJECTION

**DOI:** 10.4103/0019-5154.44805

**Published:** 2008

**Authors:** Lakshmi Chembolli, Reena Rai, Chakravarty R Srinivas

**Affiliations:** *From the Department of Dermatology, PSG Institute of Medical Sciences and Research, Peelamedu, Coimbatore - 641 004. India*

**Keywords:** *Depigmentation*, *intralesional corticosteroid*, *lymphatic drainage channels*

## Abstract

A 39-year-old female with a pruritic verrucous plaque over dorsal aspect of great toe was treated with 4 intralesional corticosteroid injections each at an interval of 2 weeks. Three months later, she developed depigmentation at the injection site and in a network-like distribution radiating away from the site. The depigmentation corresponded to the lymphatic drainage channels of the great toe.

## Introduction

Intralesional steroids are extensively used in dermatology for the treatment of keloids, lichenified hyperkeratotic lesions and numerous other conditions. The adverse effects of topical corticosteroids are listed in Table 1.^1^

**Table 1:** Adverse effects of topical corticosteroids

Systemic	Local
Suppression of hypothalamic – pituitary-adrenal axis Iatrogenic Cushing's syndrome	Epidermal atrophy – shiny, wrinkled, fragile skin with hypopigmentation, prominent vasculature, stellate pseudoscars, striae or purpura
Growth retardation in infants and children	Steroid addiction/rebound
	Glaucoma/cataracts
	Allergic or irritant contact dermatitis
	Tachyphylaxis
	Facial hypertrichosis
	Folliculitis, miliaria
	Genital ulceration
	Granuloma gluteale infantum
	Crusted (Norwegian) scabies
	Exacerbation or increased susceptibility to bacterial, fungal and viral infections
	Reactivation of Kaposi's sarcoma
	Perioral dermatitis, rosacea, acne
	Delayed wound healing

Although depigmentation is commonly observed after the injection of intralesional corticosteroids, a network of depigmentation along lymphatic channels is relatively rare. We report this case of corticosteroid-induced depigmentation along the line of lymphatics. Literature review has revealed a single report describing this side effect.^2^

## Case Report

A 39-year-old female presented with pruritic, verrucous plaques over dorsa of both big toes, which was of 2 years duration. The plaques corresponded to the strap of her footwear. They started as tiny papules that coalesced to form a hyperkeratotic plaque.

A differential diagnosis of contact dermatitis to footwear and lichen simplex chronicus was considered. Patch testing with Indian standard series showed a positive reaction to nickel, fragrance mix and balsam of Peru. Histopathology revealed orthokeratosis, parakeratosis and irregular acanthosis with minimal perivascular lymphoplasmacytic infiltrate.

**Fig. 1:**
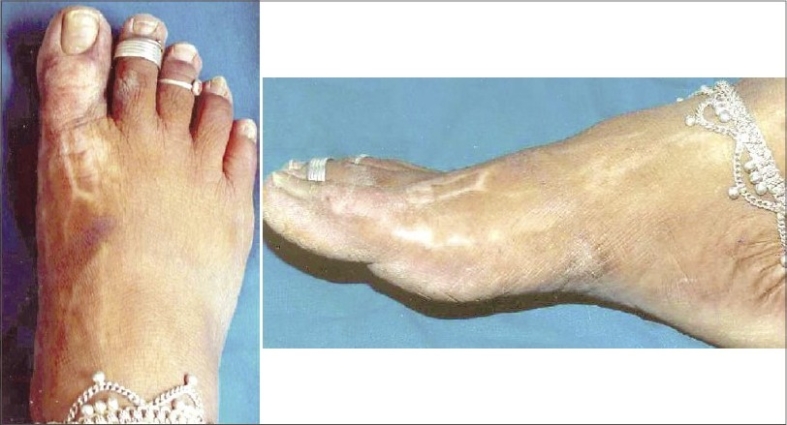
Network-like depigmentation corresponding to the lymphatic (A and B) drainage of the big toe

**Fig. 2:**
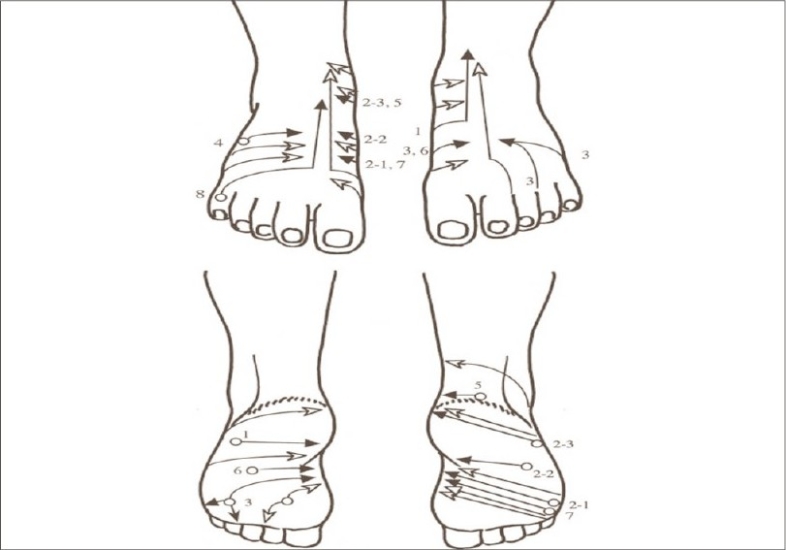
Lymphatic drainage of dorsum and sole of foot

The lesions showed initial improvement with potent topical corticosteroids, but recurred a few months later. Intralesional triamcinolone acetonide (3–4 mg) was injected. In total, 4 injections were administered at an interval of 2 weeks. Three months after the last injection, the patient reported with diffuse depigmentation at the injected site with a network-like depigmentation emanating from the injected site that became progressively less intense (Fig. 1). The depigmentation corresponded to the lymphatic drainage of the big toe (Fig. 2).^3^

## Discussion

Cells and tissues are constantly being bathed in the interstitial fluid. This compartment has an input from the arterial capillary bed and an output via both the venous capillary bed and the lymphatics. The bulk of the water, ions, and other freely diffusible and small molecules exit via the blood, while the lymph vessels remove macromolecules and large proteins and generally adopt a waste-disposal role.^4^

**Fig. 3:**
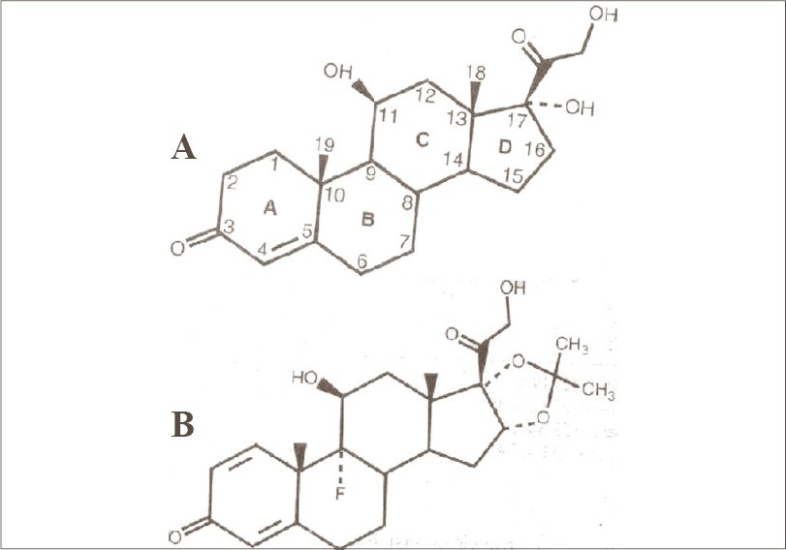
(A) hydrocortisone and (B) triamcinolone acetonide

Triamcinolone acetonide (Fig. 3) is a microcrystalline substance – a macromolecule – and is only slowly soluble; therefore, it tends to get collected in lymphatic channels. The introduction of the acetonide between hydroxyl groups at (16, 17) makes it more lipophilic with enhanced topical to systemic potency ratio. Two proteins in plasma account for almost all of the steroid binding capacity: corticosteroid-binding globulin (CBG, also called transcortin) and albumin. At normal or low concentration of corticosteroids, most of the hormone is protein bound. At higher steroid concentrations, the capacity of protein binding is exceeded, and a significant fraction of the steroid exists in free state. This unbound fraction can enter cells to mediate corticosteroid effects.^5^ Thus, the diffuse pigmentation at the injection site with a network-like depigmentation emanating away from the site is due to the retention of the corticosteroid in the lymphatics. Although the phenomenon has been frequently observed, there is only one report in literature.^2^ This article highlights depigmentation along the lymphatic channels as a side effect of intralesional steroid therapy, which is not mentioned in standard textbooks.
